# Breast Cancer Risk with Progestin Subdermal Implants: A Challenge in Patients Counseling

**DOI:** 10.3389/fendo.2021.781066

**Published:** 2021-12-17

**Authors:** Ghada Mohammed, Noha A. Mousa, Iman M. Talaat, Haya Ibrahim, Maha Saber-Ayad

**Affiliations:** ^1^ Clinical Sciences Department, College of Medicine, University of Sharjah, Sharjah, United Arab Emirates; ^2^ Department of Pathology, Faculty of Medicine, Alexandria University, Alexandria, Egypt; ^3^ Department of Medical Pharmacology, College of Medicine, Cairo University, Cairo, Egypt

**Keywords:** progestin, subdermal implants, breast cancer risk, etonogestrel, levonorgestrel

## Abstract

There is a steady global rise in the use of progestin subdermal implants, where use has increased by more than 20 times in the past two decades. BC risk has been reported with the older progestin only methods such as oral pills, injectables, and intrauterine devices, however, little is known about the risk with subdermal implants. In this review, we aim to update clinicians and researchers on the current evidence to support patient counseling and to inform future research directions. The available evidence of the association between the use of progestin subdermal implants and BC risk is discussed. We provide an overview of the potential role of endogenous progesterone in BC development. The chemical structure and molecular targets of synthetic progestins of relevance are summarized together with the preclinical and clinical evidence on their association with BC risk. We review all studies that investigated the action of the specific progestins included in subdermal implants. As well, we discuss the potential effect of the use of subdermal implants in women at increased BC risk, including carriers of BC susceptibility genetic mutations.

## Introduction

Hormonal contraception is widely used by women of reproductive age, and its association with breast cancer (BC) risk has been investigated for decades. This risk has been extensively studied for estrogen-containing contraceptives which provides grounds for counseling women contemplating combined contraception (1–4). Meanwhile, conflicting or inadequate data has been published on progestin-only contraceptives (5).

The progestin-subdermal implants are increasingly popular progestin-only contraceptives since the first implant was licensed in 1983. They provide a highly effective, long-acting and reversible method of contraception (6). According to the National Institute for Health and Care Excellence (NICE), subdermal implants have a very low failure rate (less than 1 pregnancy per 1000 implants fitted over 3 years) (7). With their increasing popularity, clinicians are facing a challenge in counseling patients about the risk of BC associated with these methods. The current clinical guidelines do not provide adequate information to support clinicians or patients to make decisions regarding the use of these methods.

In this review, we aim to update clinicians and researchers on the current evidence to support patient counseling and to inform future research directions. All found references on the association of progestin subdermal implants with BC risk were included in this review. As well, key studies (pre-clinical and clinical) on BC risk with endogenous progesterone and other progestin-containing contraceptives are overviewed. In addition to published research studies and clinical guidelines, we have also used the PubChem and the Human Protein Atlas databases to identify proteins (including receptors) for which progestins and progesterone have a high binding affinity.

## Endogenous Progesterone in Normal Breast Tissue

Progesterone exerts its main physiological role by binding to the progesterone receptor (PR). The function of PR in the normal breast has been extensively studied (8). The resulting hormone-receptor complex initiates transcription of the target PR gene. This gene uses two specific promotors and starts sites for translation in the first exon to generate several transcripts, including protein-coding, and non-protein-coding transcripts. PR has two isoforms, PR-A and PR-B, derived from the same gene but induced by two different promoters, resulting in two sets of transcripts (9). Progesterone-dependent promoters were also shown to induce SRC-dependent MAPK signaling upon stimulation by progesterone (10). In addition, progesterone was shown to have a stimulatory effect on mitochondrial membrane potential and cellular respiration that can result in reduced apoptosis (11).

It is important to note that many, but not all of progesterone cellular actions are mediated through estrogen. They together coordinate the development of the ductal and lobular systems of the breast. Accordingly, there is a great challenge in identifying the individual actions of progesterone independently from those of estrogen (12, 13). Other factors can also add to such challenges including the receptor-independent effects of progesterone, its overlapping roles with androgens and prolactin, and the considerable variation of progesterone level during the menstrual cycle, pregnancy and perimenopause (14).

## Endogenous Progesterone in BC

Various roles were assumed for the contribution of progesterone in BC development and progression (5, 15). These include immunomodulatory roles, paracrine stimulation of BC stem cells and the effect of progesterone metabolites (16–18). 4-pregnanes and 5α-pregnanes are examples of progesterone metabolites that were found to affect breast tissue. They are synthesized in different tissues including ovaries, adrenals and adipose tissue. 5α-pregnanes can induce BC cell proliferation *in vitro* and is more abundant in BC tissue compared to normal breast tissue, whereas 4-pregnanes may have a suppressive effect on proliferation (19). However, little has been done to explore the clinical value of measuring circulatory progesterone metabolites or their exact correlation with BC risk and prognosis (5).

PR-B is currently recognized for its role in breast carcinogenesis, and it appears to be involved in the regulation of more genes in BC cells than PR-A. Furthermore, it mediates cell cycle progression through extranuclear signalling, unlike isoform A which is limited to the nucleus (20, 21). Moreover, knocking out the PR-B isoform in the mammary mouse model led to abnormal mammary gland development which did not occur when PR-A was knocked out (22). There is lacking data on the preferential binding of synthetic progestins to either PR-A or PR-B. However, Schindler et al. suggested that no differences are expected to show since both isoforms have identical steroid-binding domains (23).

Based on the available evidence that progesterone possibly plays a role in BC, the potential therapeutic effect of anti-progesterone medications were studied (24). Despite the proof of principle, those studies remain limited hindering their wide clinical use as adjunct endocrine therapies or as chemo-preventive agents. Noteworthy, recent expert views - as thoroughly discussed by Horwitz and Carol- are proposing that endogenous progesterone should not be considered a breast carcinogen in the case of the normal breast (25).

## Progestins in Clinical Use: (Structure and Function)

Progestins or progestagens are terminologies often used alternatively to describe synthetic exogenous compounds with progesterone-like action. Progestins are generally classified into C-21 compounds (chemically close to the structure of endogenous progesterone with 21 carbon atoms) and C-19 compounds (chemically close to the structure of testosterone with 19 carbon atoms) (26).

The most common progestin preparations are those used as contraceptives. Progestins are also used as a key component of hormone replacement therapy (HRT) (27). The proven anti-proliferative effect of progesterone on the endometrial tissue reduces the risk of endometrial hyperplasia and endometrial cancer in women using such therapy (28). However, there is no evidence that such a favorable effect is maintained in the case of the breast tissue. In addition, progestins are widely used during early pregnancy in patients with threatened miscarriage and patients undergoing assisted reproduction as it is believed to provide luteal phase support and hence reduces the risk of early pregnancy loss (29, 30).

## Progestin Subdermal Implants

### Overview and Structure

Multiple subdermal implants, with different progestin types, were initially introduced in different countries, however, only a few are currently commercially available for use worldwide, while others have been either discontinued or in limited use (31, 32). These most popular subdermal implants contain one of two progestins: levonorgestrel (LNG) or etonogestrel (ENG) as outlined in **Box 1**. Detailed structural differences can be reviewed in **Table 1**, as outlined in the PubChem database (https://pubchem.ncbi.nlm.nih.gov/). Of note, ENG is an active metabolite of desogestrel, which has been more widely used in oral formulations. In this review, we focus on these two implants, both shown to have comparable effectiveness, release characteristics and pharmacokinetics (6).

Box 1 **Progestin Subdermal Implants in Commercial Use.**

**Table:** 

Progestin type	Generic name	Duration of effect
*LNG*	Norplant	5 years
*LNG*	Jadelle	5 years
*LNG*	Sino-implant2	4 years
*ENG*	Implanon	3 years
*ENG*	Nexplanon	3 years

**Table 1:** Chemical structure of progestins included in subdermal implants.

Progestin	Chemical formula	Detailed structure
Progesterone 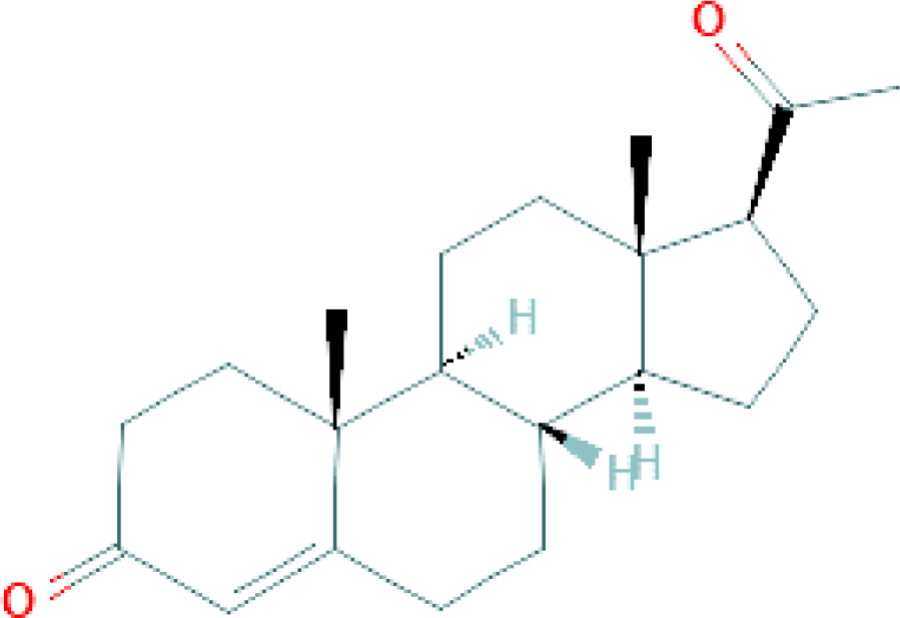 Progesterone is a C21-steroid hormone in which a pregnane skeleton carries oxo substituents at positions 3 and 20 and is unsaturated at C (4)-C. It is a 20-oxo steroid, a 3-oxo-Delta steroid and a C21-steroid hormone (33)		
Levonorgestrel(Oral pills, IUS, Implants)	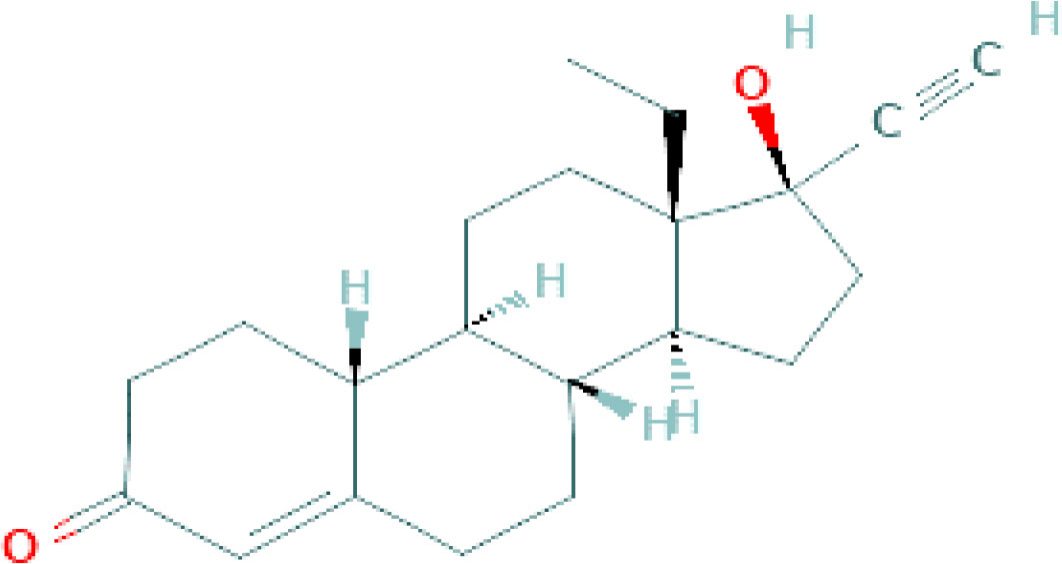	A 17-betahydroxy steroid, a 3-oxo-Delta (4) synthetic progestogen and levorotatory form of norgestrel. It displays progestational and androgenic activity, but it lacks estrogen-like activity. Levonorgestrel binds to the progesterone receptor in the nucleus of target cells (34).
Desogestrel(Oral pills)	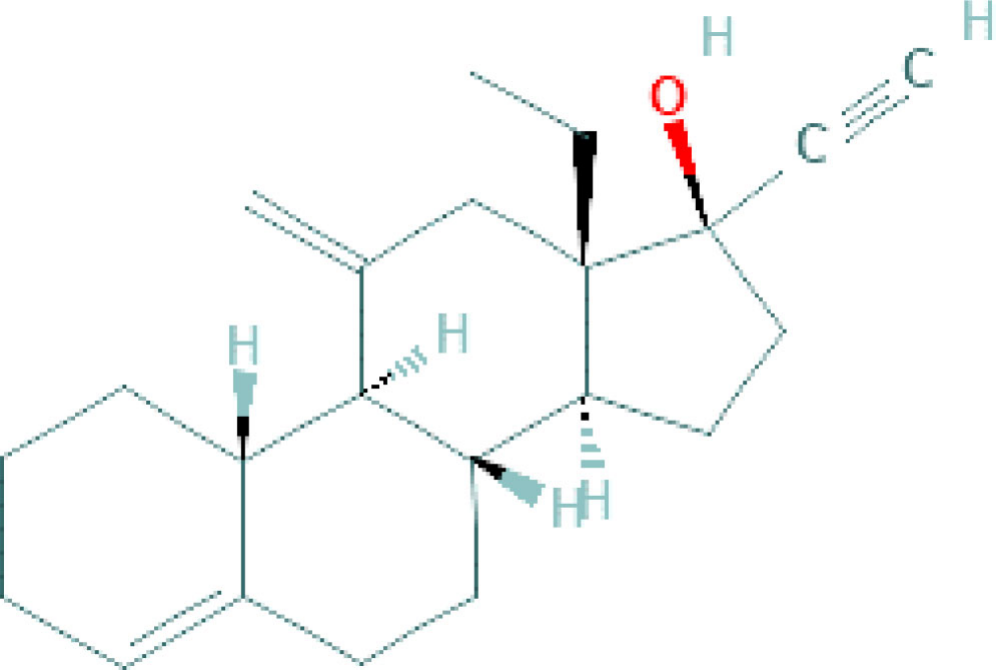	Desogestrel, a semi-synthetic compound and a prodrug, is a third-generation progestogen and hence, a member of the gonane family. It is a 17 beta-hydroxy synthetic progestogen structurally related to levonorgestrel, with PR agonistic activity (23)
Etonogestrel(Subdermal implants)	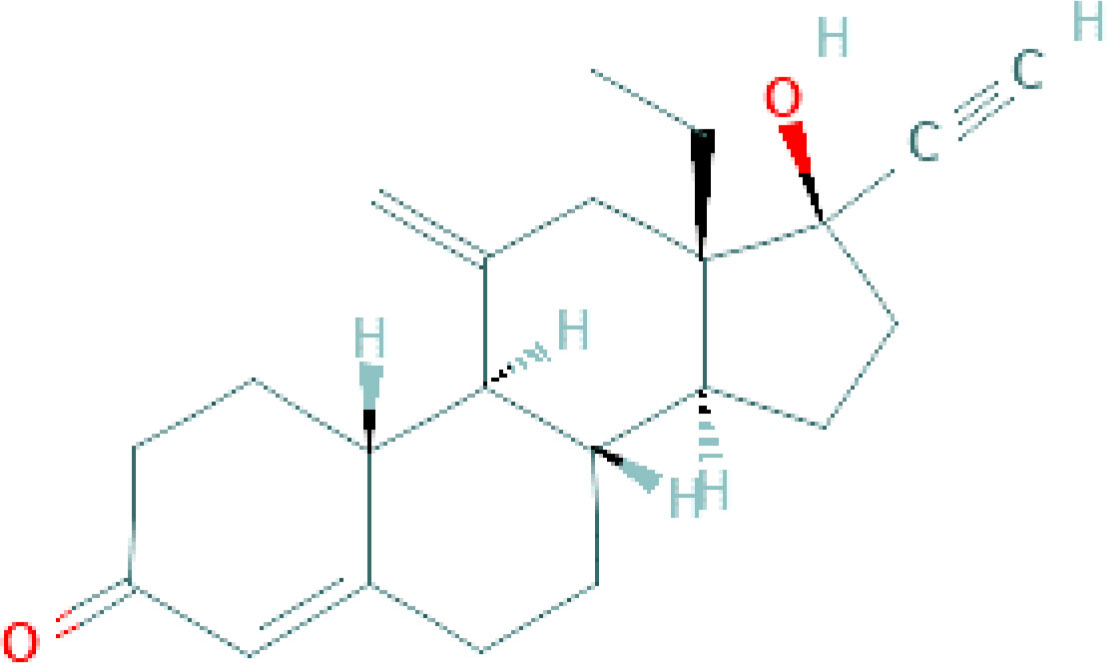	Etonogestrel molecule is a 3-keto-desogestrel or 19-nortestosterone which is a synthetic biologically active metabolite of desogestrel (35)

### Identification of Cellular Targets of Progestins in Subdermal Implants

**Figure 1:**
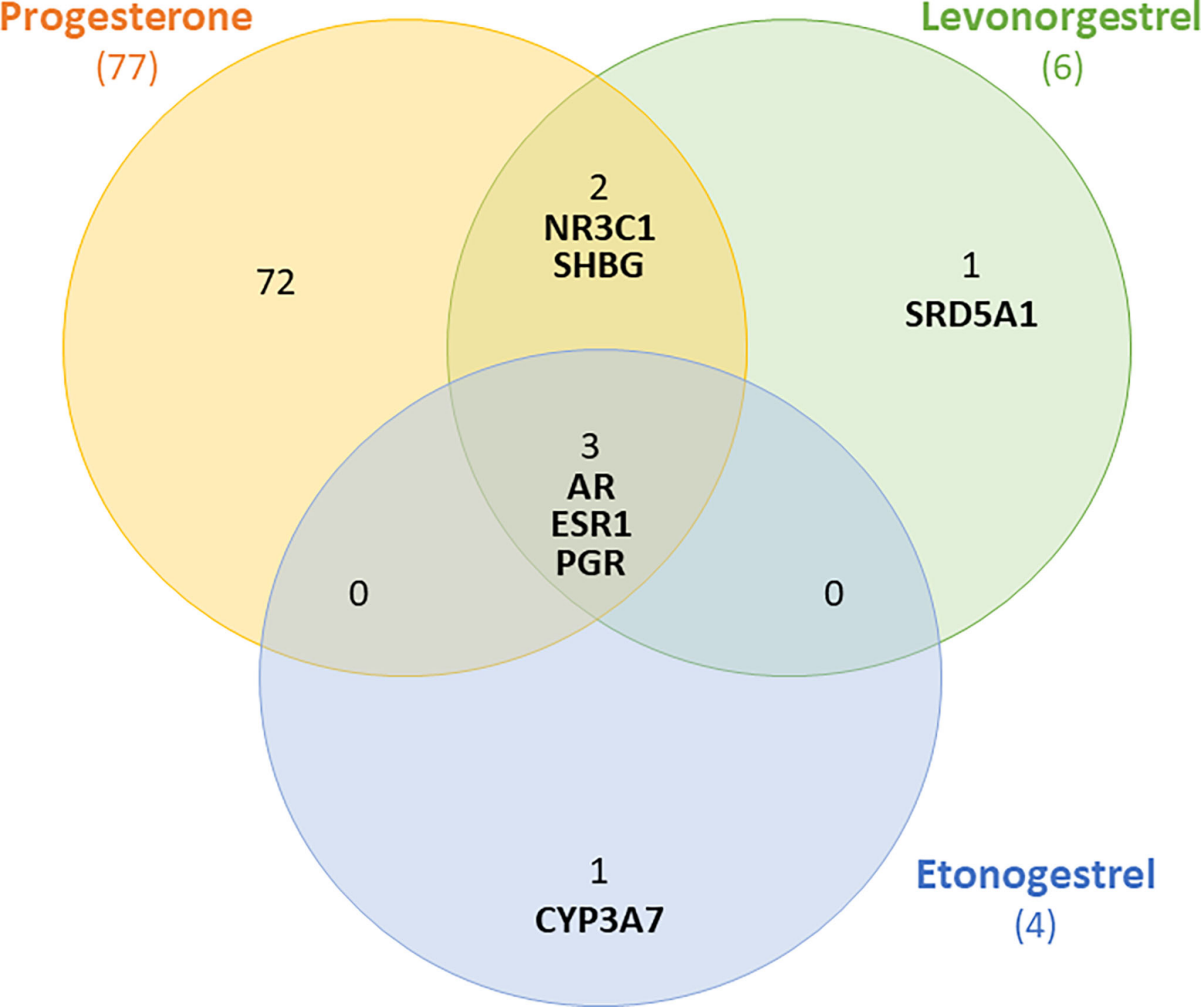
Venn diagram showing receptors to which levonorgestrel, etonogestrel and progesterone can bind with high affinity. Shared receptors include androgen (AR), estrogen-1(ESR1) and progesterone receptors (PGR).

To understand how progestins in subdermal implants may differ from endogenous progesterone in terms of potential breast carcinogenesis, it is necessary to identify their main cellular targets. We used the PubChem database to identify the proteins (including receptors) for which LNG, ENG, and progesterone have a high binding affinity (36). In common, the three compounds bind to the progesterone receptors (PGR), androgen (AR), and estrogen-1 (ESR1) as outlined in the following Venn diagram (**Figure 1**).

In addition, we searched the human protein atlas (http://www.proteinatlas.org/, tissue atlas section) (37) to retrieve the expression of proteins highly expressed in the normal breast tissue to which progesterone has a high binding affinity (38). Out of those proteins, progesterone mainly binds to 3 proteins, namely; TARDBP, MAPK1 and NR3C1. LNG also binds, with high affinity, to NR3C1. Using the STRING protein functional analysis network (39), annotations of these proteins and the RNA expression of other proteins with progesterone high binding affinity are summarized (**Table 2** and **Supplemental Figures S1**–**S9**).

**Table 2:** Annotations of proteins to which progesterone have a high affinity.

Protein Expression	Annotation
TARDBP	TAR DNA-binding protein is DNA and RNA-binding protein, regulating transcription and splicing It may be involved in the synthesis of microRNA, apoptosis and cell division (40).
MAPK1	Mitogen-activated protein kinase 1 is also a Ser/Thr-kinase and an essential component of the MAP kinase signal transduction pathway. The MAPK/ERK cascade exerts a contextual role depending on the cellular environment. It mediates a variety of biological activities such as cell growth, adhesion, survival, and differentiation (41).
NR3C1	Nuclear Glucocorticoid receptor. It has transcriptional repression activity. It has the least effect on transcriptional activation, compared to the activity of all isoforms (42).
**RNA Expression**	**Annotation**
NME2	Nucleoside diphosphate kinase; NME2 has a major role in the synthesis of nucleoside triphosphates (GTP, CTP and TTP but not ATP). It suppresses Rho activity by interacting with AKAP13/LBC. Rho (GTPases) are indirectly linked to cancer by their interaction with known oncogenes (e.g. Raf and Ras), (43). NME2 also acts as a transcriptional activator of the MYC gene.
PTEN	Phosphatase and tensin homolog; It is a tumor suppressor with a lipid phosphatase activity. Inactivation of PTEN lipid phosphatase leads to the interaction of the Hippo and PI3K/Akt pathways, thus promoting tumorigenesis (44).
CDKN1B	Cyclin-dependent kinase inhibitor 1B is one of the key regulators of the progression of the cell cycle. It inhibits the kinase activity of CDK2 bound to cyclin A, leading to G1 arrest. It also markedly inhibits cyclin E- and cyclin A- CDK2 complexes (45).
RXRA	Retinoic acid receptor RXR-alpha. Such receptors bind as heterodimers to their target response elements in response to their ligands to regulate gene expression (46).
OXTR	Oxytocin receptor, a G protein-coupled receptor that mediates various effects of oxytocin (47).
CDK4	Cyclin-dependent kinase 4 is Ser/Thr-kinase component of cyclin D-CDK4 (DC) complexes. It phosphorylate and inhibit members of the retinoblastoma (RB) protein family including RB1 and regulate the cell-cycle during G (1)/S transition. Cyclin D-CDK4 complexes play a key role in cell cycle progression (48).

Relevant to the discussion on cellular targets, both LNG and ENG have an affinity to AR (as shown in **Figure 1**). The AR is expressed in 70–90% of BC tissue samples (49). However, in BC patients there is ongoing controversy on the paradoxical role of AR (50). It appears that AR has a tumor suppressor effect (51), mostly in ER-positive BC, whereas it plays an oncogenic role in ER-negative BC, mainly mediated by FOXA1 (52, 53). This data adds to the complexity of predicting the effect of any pharmacologic agent with a high affinity to AR since we would not be certain if its pro or anti-tumor role will be activated in a normal breast. Nevertheless, a case-control study of women with benign breast conditions showed no correlation between AR expression in the normal breast tissue and subsequent risk of developing BC (54). This finding may be slightly assuring, however, it did not directly examine the effect of exogenous androgens on BC risk in healthy women.

## Progestin-Only Contraception and BC risk

### Evidence From Pre-Clinical Studies

The notion of progestins to initiate BC tumors appears to be derived from early experimental toxicity studies (2, 55). For instance, Depot Medroxy Progesterone Acetate (DMPA) has been tested for its carcinogenic effect in mice, where it induced mammary adenocarcinomas and in dogs where it induced mammary hyperplasia (56, 57). Based on a review of the FDA records, LNG has been evaluated for its BC risk in animal studies with no evidence of carcinogenicity. Different doses of LNG were tested in beagles and rhesus monkeys for several years without evidence of inducing mammary gland malignancies. However, evaluation of the long-term toxicity of desogestrel on mice and rats showed a higher rate of mammary adenocarcinoma associated with higher doses (55).

### Evidence of the Effect of Progestins on Mammographic Breast Density

Mammographic breast density (MBD) is recognized as an independent marker for BC risk (58, 59). Moreover, MBD appears to be a modifiable risk factor in response to reproductive and lifestyle modifications (60, 61), as well as to hormonal and anti-hormonal therapies. Therefore, it has been used as an intermediate marker to evaluate the effect of risk-increasing (62, 63) and risk-reducing interventions (64–67). As a result, MBD has been often used to evaluate breast safety of newly introduced hormonal therapies, such as selective estrogen receptor modulators (SERM), selective progesterone receptor modulators (SPRM), aromatase inhibitors, gonadotropin-releasing hormone (GnRH) agonists, and phytoestrogens (68).

In postmenopausal women, there is substantial evidence that progestin-containing HRT increases MBD in comparison to estrogen alone HRT or placebo (69–71). In reproductive age and premenopausal women, multiple studies investigated the effect of combined estrogen and progestin contraceptives on MBD (72). However, when it comes to the use of progestin-only contraceptives in reproductive age women, limited quality evidence indicated variable outcomes of their effect on MBD. For example, the use of the levonorgestrel intrauterine system (LNG-IUS) led to an increased MBD after one and a half years in one report (73), whereas the discontinuation of DMPA injection led to increased MBD in another report (74). A similar MBD-reducing effect was observed in a small study of 26 women who were given micronized progesterone in the luteal phase for 6 months (75). There have been no studies on the effect of progestin-containing subdermal implants on MBD. Such scarce evidence is difficult to interpret or to further rely on during patient counseling. The extrapolation from postmenopausal studies or from studies that included combined estrogen and progesterone would not be optimally valid.

### Evidence From Clinical Studies

In postmenopausal women, the long term use of progestin-containing HRT was associated with increased BC risk (76). In women of reproductive age, progestin-only contraceptives including oral pills, injectables, intrauterine devices and implants, were evaluated for their association with BC risk in multiple large-scale clinical studies with variable outcomes as summarized in **Table 3** (1, 77–79, 83).

**Table 3:** Summary of key evidence on BC risk with progestin-only contraceptives.

Progestin	BC Risk Evidence
**Progestin Only Pills (POPs)** ***various progestin types***	No significant risk OR 0.9 (95% CI not reported) (77) RR 1.1 (95% CI: 0.8–1.7) (78)
**Depo Medroxy Progesterone Acetate (DMPA) injectable**	No significant risk (1, 79) Increased risk (RR 2.2) (80)
**Levonorgestrel Intrauterine system (LNG-IUS)** **Progestin-only subdermal implants (LNG or ENG)**	Increased risk (recent studies) RR 1.21 (95% CI, 1.11 to 1.33) (1) RR 1.19 (95% CI, 1.13–1.25) (81)
	8-fold increased risk (82) (*older study, limited sample*) No significant risk (1, 79) *(recent studies, relatively limited sample)*

Various Progesterone-Only Pills (POPs) have been investigated for their association with BC risk. Norethindrone, levonorgestrel and desogestrel POPs are the most widely studied. In the Norwegian-Swedish Women’s Lifestyle and Health Cohort Study (including 103,027 women), the exclusive ever use of POPs was not associated with increased BC risk, while the current or recent use of POPs was found to increase the risk of BC (78). Mørch and colleagues conducted a nation-wide analysis of the Danish registries which included 1.8 million women throughout 11 years of follow-up. They investigated BC risk in relation to current and previous hormonal contraceptive use. Compared to never using hormonal contraception, levonorgestrel-containing POPs were associated with significantly increased BC risk (RR: 1.93). However, norethindrone and desogestrel-containing POPs were not associated with increased risk of BC in the same study (1). Likewise, the progestin-only injectables in the form of DMPA were found in some case-control studies to be associated with an increased risk of invasive BC (80). Other studies did not confirm this association (1, 79).

The LNG-IUS is a highly popular effective method of contraception and is also widely used for the management of menorrhagia. It releases a minute daily dose of LNG into the uterine cavity leading to high LNG concentration in the endometrium (84). For a long time, LNG-IUS has been perceived by physicians as a local method of a very minimal systemic effect. Earlier studies reported no evidence of increased BC risk with using this method (85, 86) and it was included in studies of BC patients (87). However, recent evidence demonstrated an association of LNG-IUS and increased BC risk (A relative risk of around 1.2 was reported) (1, 81). This risk can be explained by the evidence of significant systemic LNG concentration, reaching roughly half the level achieved with oral formulations (88, 89). Yet, these studies carry limitations including the relatively small number of users and the consideration of the confounding factors such as the prior use of combined oral contraceptives (COCs) (90).

When it comes to progestin-only subdermal implants, scarce data is available on their association with BC risk. Thus far, there has been no dedicated clinical study to evaluate this risk with subdermal implants. However, a few studies included users of subdermal contraceptive implants among other types of contraception. In the study by Mørch and colleagues, nine BC events were found out of 42,217 person-years of using subdermal implants, with an insignificant relative risk of 0.93 (1). Similarly a case control study including 4,575 women with BC and 4,682 controls, evaluated the usage of progestin injectables and progestin implants with BC risk. Five of twelve women who used progestin implants developed BC, however, this was not a statistically significant risk (79). Nevertheless, in a large case-control study conducted in the United States, where less than 1% of the study population used subdermal implants, the risk of BC was found to be increased by more than 8-fold in the implant users (82). Apparantly, all these studies included a limited number of implant users relative to the other studied hormonal contraceptives.

In regards to the effect of dose and duration of progestin-only contraceptives on BC risk, an increased risk of BC was suggested in some studies (82), while not confirmed by others (1). For the LNG-IUS, the reported increase in BC risk by Mørch and colleagues was interestingly not affected by the duration of use. Nevertheless, an earlier nation-wide study of a Finnish cohort by Soini and colleagues suggested an increased risk with the extended duration of use of LNG-IUS (81). Such inconsistency adds again to the difficulty in counseling patients on the safe duration of use. For subdermal implants, there is no data available on the dose or duration-response relationship.

## Progesterone and BC Susceptibility Genetic Mutation Carriers

An important study by Yongxian Ma et al. showed that BRCA1 inhibits the PR activity and the knock-out of the BRCA1 gene could enhance the progesterone-stimulated PR activity. This indicates that BRCA1 is an important suppressor of the PR activity and its mutated form is not able to maintain such suppression (91). BRCA1 and 2 mutation carriers seem to additionally have an altered PR expression in breast tissue compared to non-carrier women. There is evidence of the loss of the B isoform leaving predominance to the A isoform, which led to premalignant lesions and invasive BC disease (92). Furthermore, the breast tissue of BRCA1 mutation carriers may have a different organization or composition of the extracellular matrix (93). Taken together, these studies may suggest that the response of BRCA 1 and 2 mutation carriers to exogenous progesterone may be exaggerated. In clinical terms, those women may be hypersensitive to progestin preparations in general.

In the context of clinical studies, interestingly, there is evidence that BRCA1/2 mutation carriers could be exposed to significantly higher levels of serum progesterone during the luteal phase of the menstrual cycle (reported to be around 121% higher than in matched control women) (94). In addition, using progestin-containing HRT in BRCA1 mutation carriers who underwent prophylactic oophorectomy carried an increased BC risk compared to using estrogen alone HRT (22% vs 12% cumulative risks) (95). This is in line with the findings of the Women’s Health Initiative (WHI) study in non-carrier women (76). Combined oral contraceptive pills, were also found to increase the risk of BC in BRCA1 mutation carriers, especially if used more than 5 years or in younger women less than 30 years old (96). Similar findings were shown with both BRCA1 and BRCA2 mutation carriers who ever used combined oral contraceptive pills. Likewise, since there are no studies of the effect of progestin-only containing subdermal implants, it remains difficult to accurately counsel those young patients on the safety of these contraceptives. The UK Medical Eligibility Criteria for Contraceptive Use (UKMEC) considers subdermal implants and other progestin-only contraceptives as category 2 in BRCA1/2 mutation carriers *(“UKMEC Category 2: A condition where the advantages of using the method generally outweigh the theoretical or proven risks*”) (97). However, both the pre-clinical and clinical studies mentioned above do not seem to support this categorization. Until more conclusive evidence exists, we may suggest that clinicians should adopt a cautious approach towards prescribing progestin-containing contraceptives in this special group of women who already have a very high baseline BC risk.

## Clinical and Research Evidence for Patient Counseling

Limited evidence is available on the risk of BC associated with contraceptive implants. The available limited number of studies, showed no significant increased risk. However, if the risk of BC associated with the LNG-IUS is considered, it is difficult to completely dismiss the possibility of a comparable risk with the LNG and ENG implants. A recent review has addressed the counseling of women on the risk of BC with hormonal contraceptives, however, did not include subdermal implants (98).

The Faculty of Sexual and Reproductive Healthcare (FSRH) published recent guidelines on the ENG contraceptive implant. They suggested no significant increase risk of BC, however, they highlighted the limited available data to exclude such an association (99). The recently revised practice advisory by the American College of Obstetricians and Gynecologists (ACOG) analyzed the outcomes of the large cohort study by Mørch and colleagues and other evidence on the risk of BC with hormonal contraceptives. In brief, the ACOG concludes that BC risk with progestin-only contraceptives remains inconsistent. There was no observed increased risk with contraceptive implants, while the risk with LNG-IUS was comparable to that of oral contraceptives. However, the ACOG has also highlighted the limitations of the study and the difficulty in justifying the absence of risk related to dosage and duration of use (100). The ACOG presented a few points of advice to guide physicians while counseling women on BC risk with hormonal contraceptives. However, they did not specify any advice on subdermal implants.

## Conclusion

There is an obvious lack of clinical guidelines or solid research data on the safety of progestin subdermal implants. This left physicians with a challenge in providing counseling for women interested in starting these methods, or current users who consider using them long-term. A shared decision between the physician and the patient should highlight our inability to guarantee BC relevant safety. Weighing benefits versus risks for each patient based on her own baseline BC risk seems a valid approach at this stage. Hereditary and acquired risk factors (e.g. reproductive factors and obesity) that may potentiate any small added risk using these methods should be considered. Since all previous studies did not focus on subdermal implants, conclusions will remain controversial until dedicated well-designed studies are conducted.

## Author Contributions

GM wrote the first draft, edited, and reviewed the manuscript. NM contributed to the concept, edited, and review the manuscript. MS-A contributed to specialized sections, editing, and review of the manuscript. IT contributed to specialized sections of the manuscript. HI contributed to searching and presenting protein atlas data. All authors contributed to the article and approved the submitted version.

## Conflict of Interest

The authors declare that the research was conducted in the absence of any commercial or financial relationships that could be construed as a potential conflict of interest.

## Publisher’s Note

All claims expressed in this article are solely those of the authors and do not necessarily represent those of their affiliated organizations, or those of the publisher, the editors and the reviewers. Any product that may be evaluated in this article, or claim that may be made by its manufacturer, is not guaranteed or endorsed by the publisher.
