# Supplementary material for: Breast Cancer Risk with Progestin Subdermal Implants: A Challenge in Patients Counseling
Source: Front Endocrinol (Lausanne). 2021 Dec 17;12:781066. doi: 10.3389/fendo.2021.781066 (PMC8719328; doi:10.3389/fendo.2021.781066)
Supplement: Supplementary file 1 [file Presentation_1.pptx]

## Slide 1
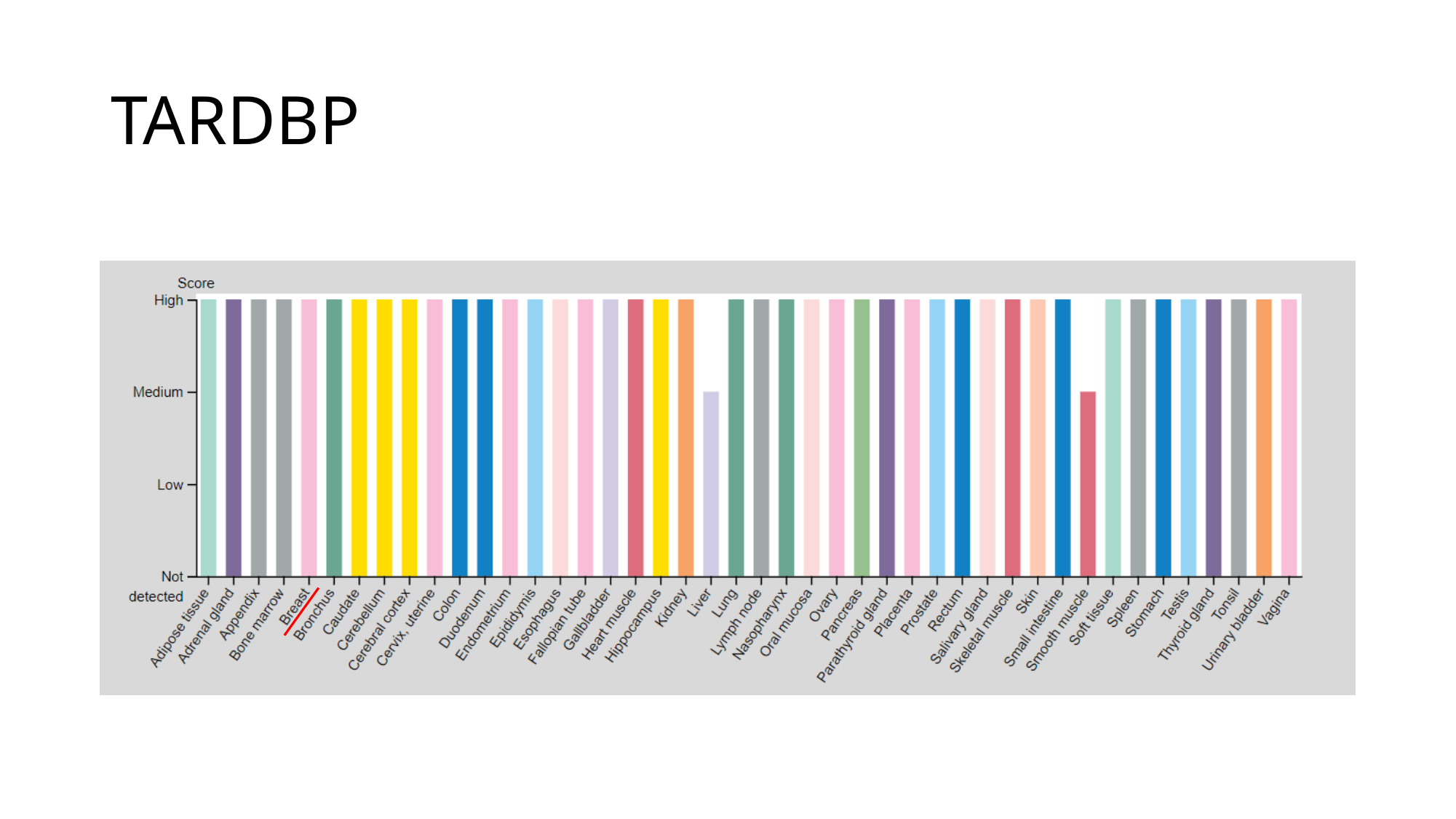

# TARDBP

## Slide 2
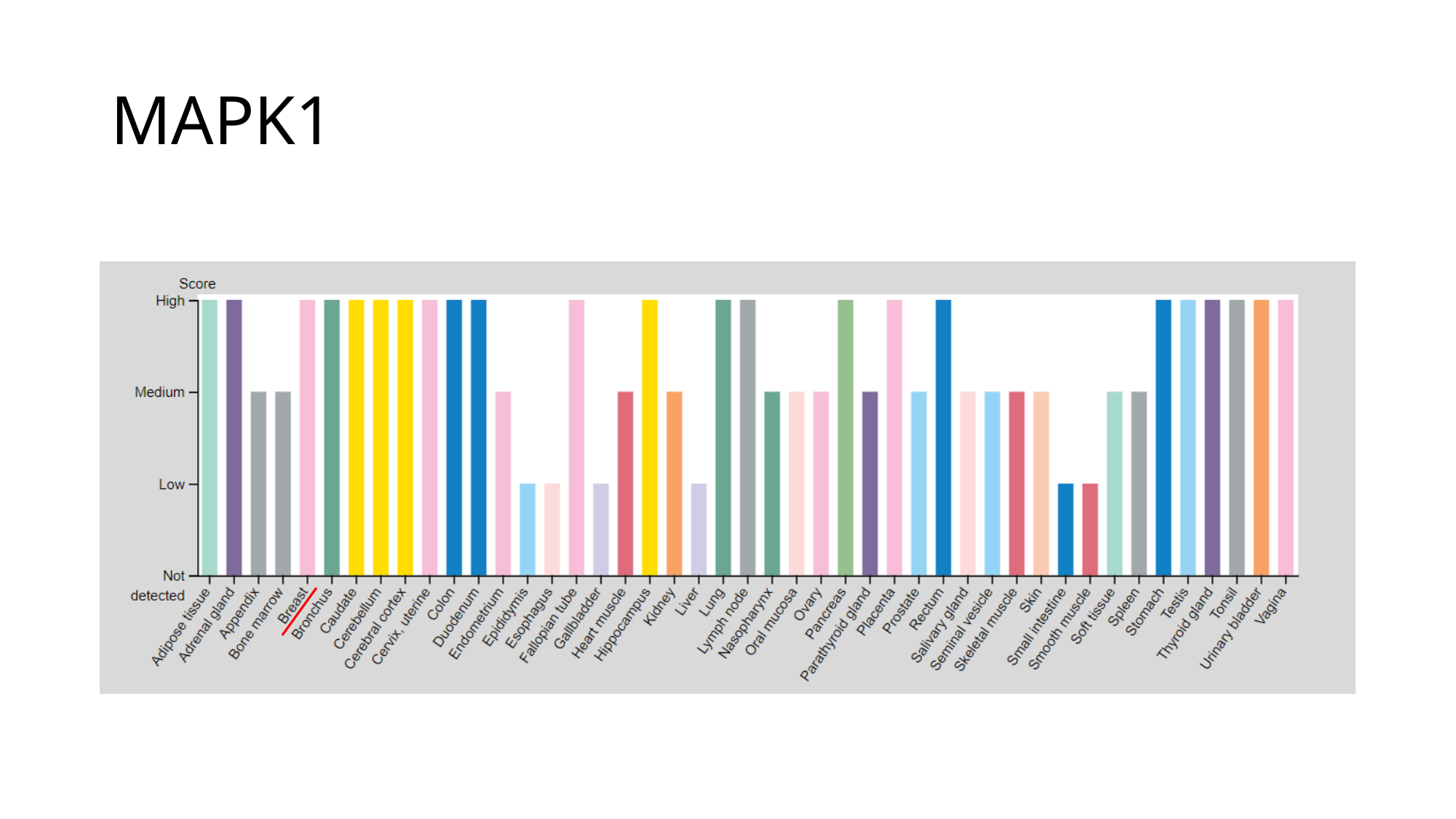

# MAPK1

## Slide 3
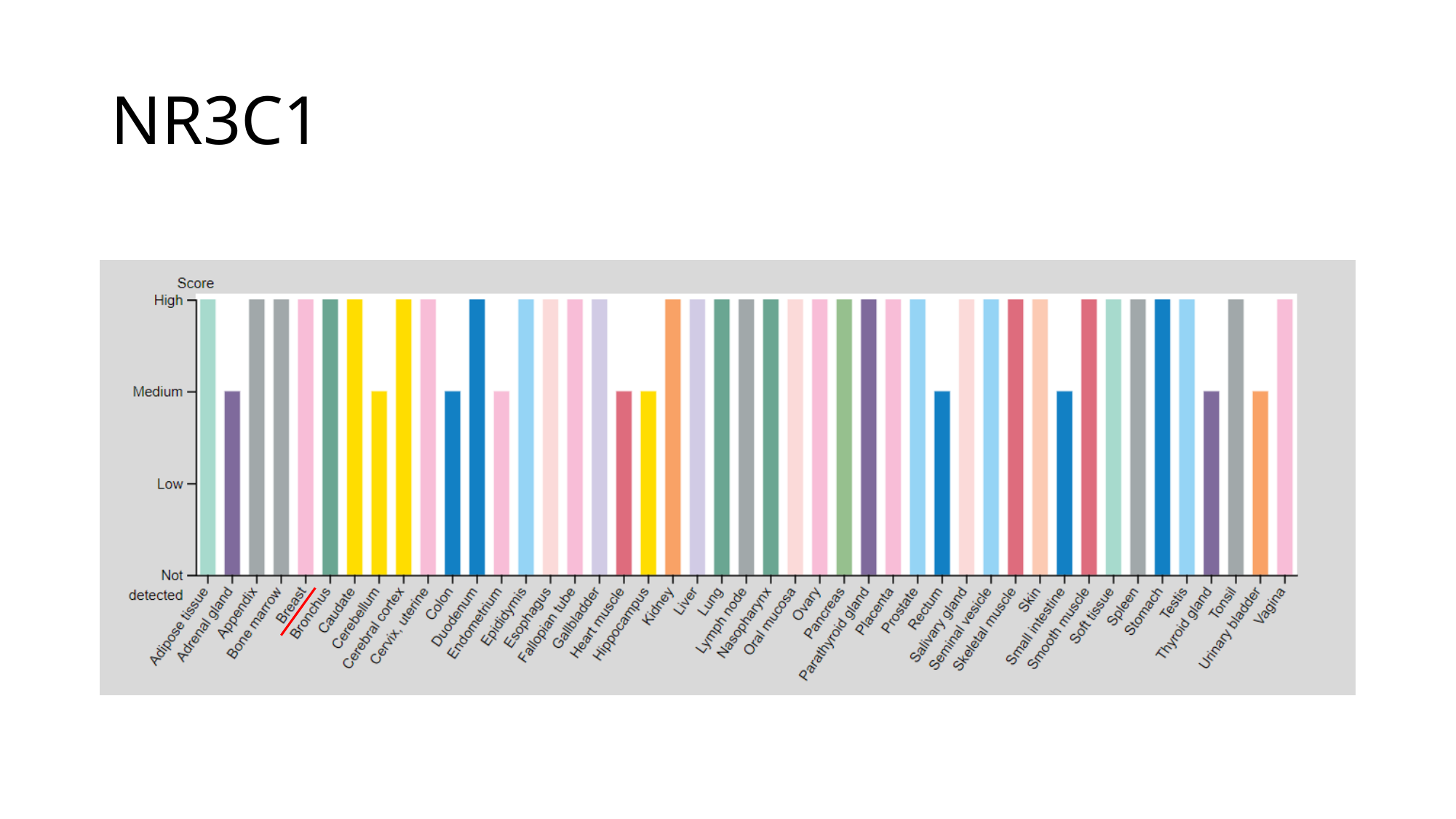

# NR3C1

## Slide 4
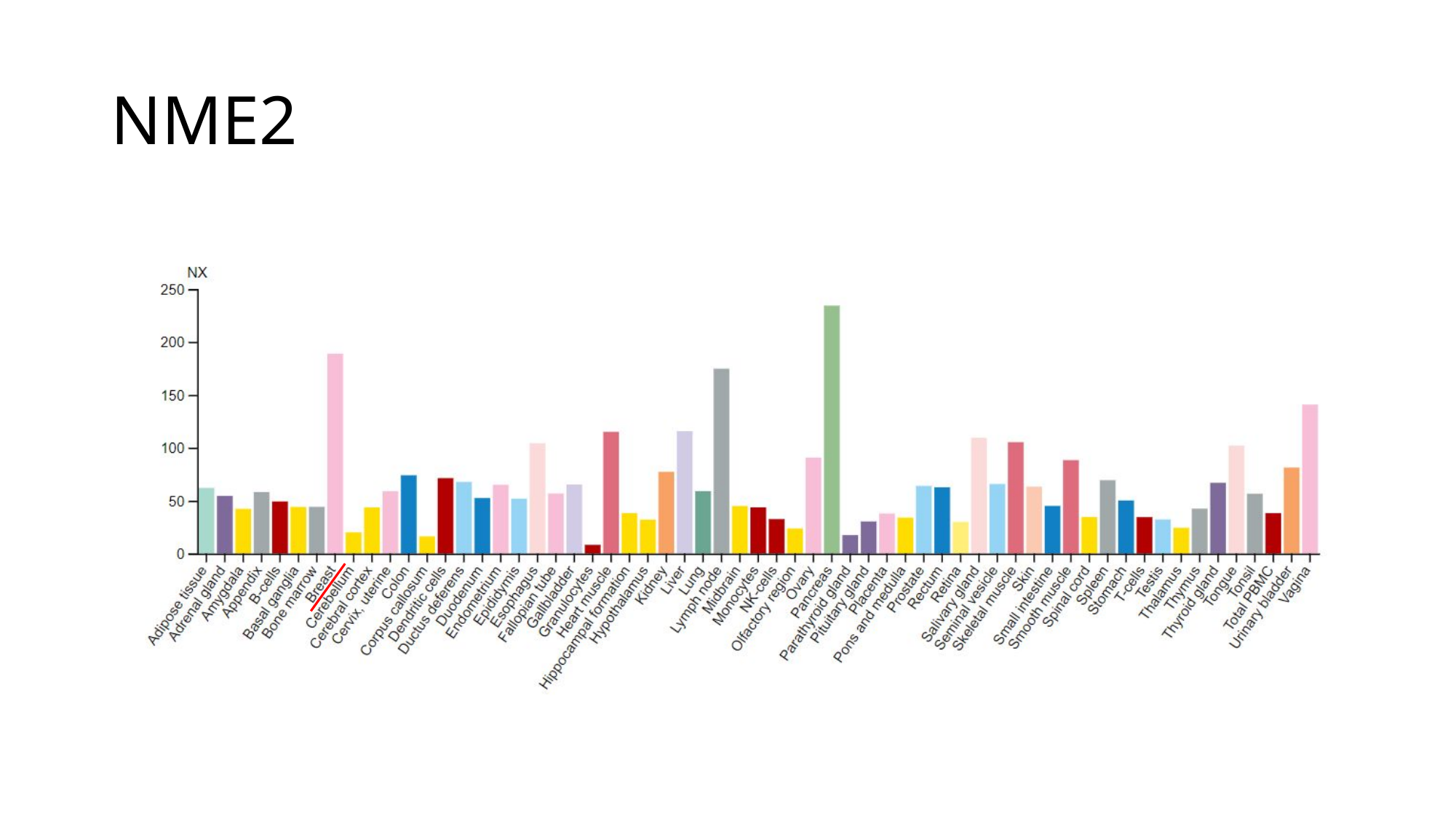

# NME2

## Slide 5
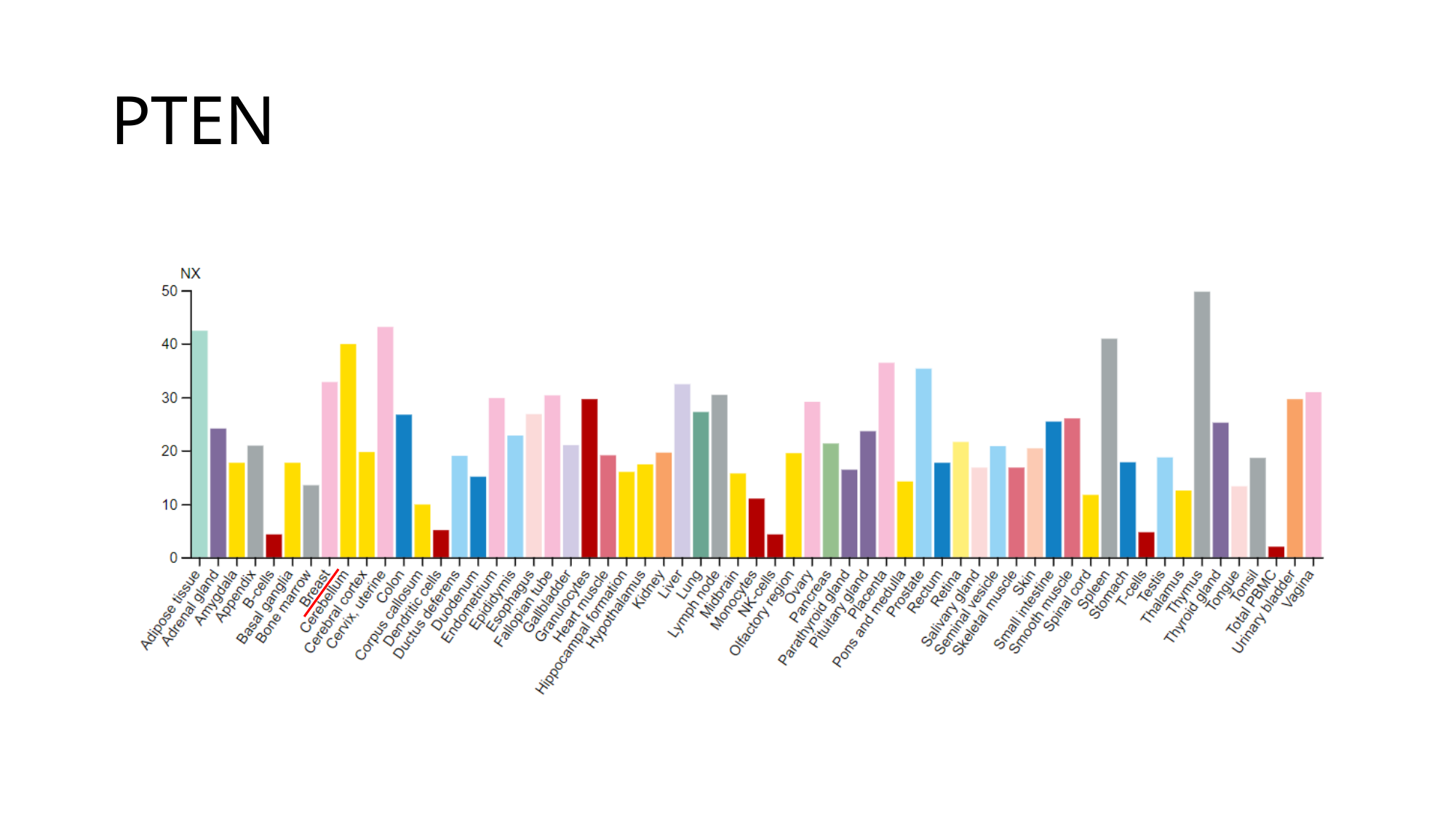

# PTEN

## Slide 6
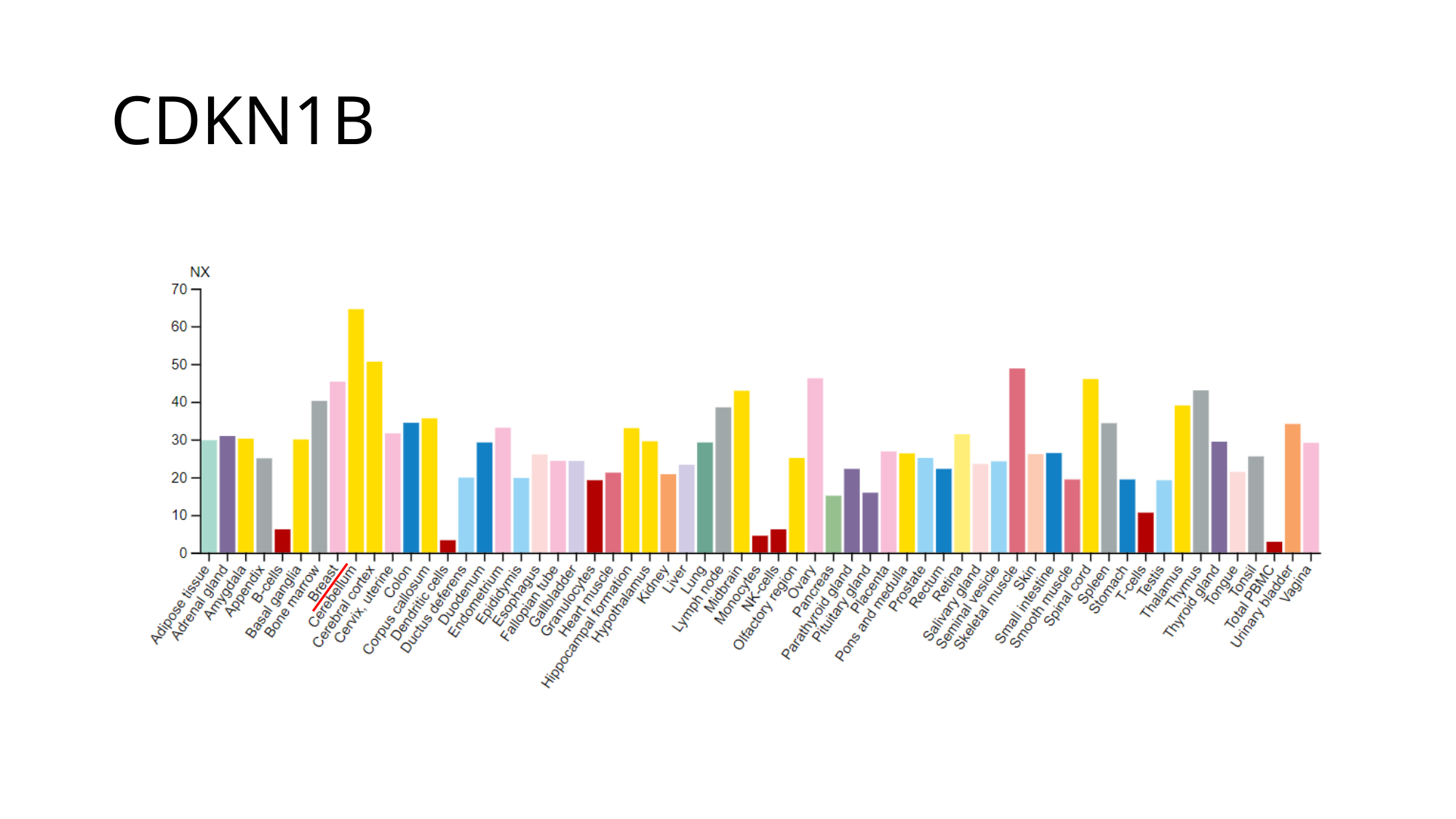

# CDKN1B

## Slide 7
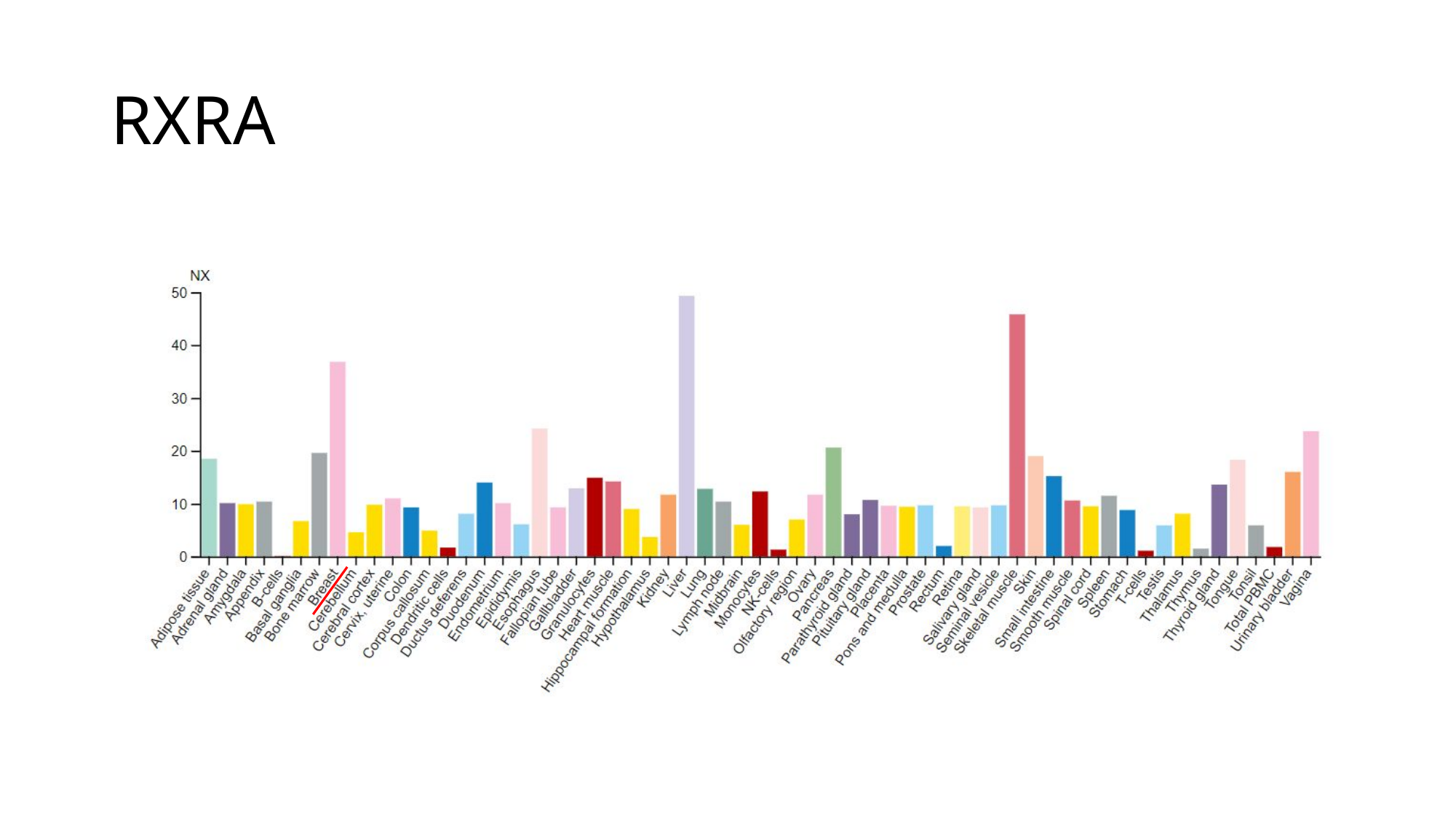

# RXRA

## Slide 8
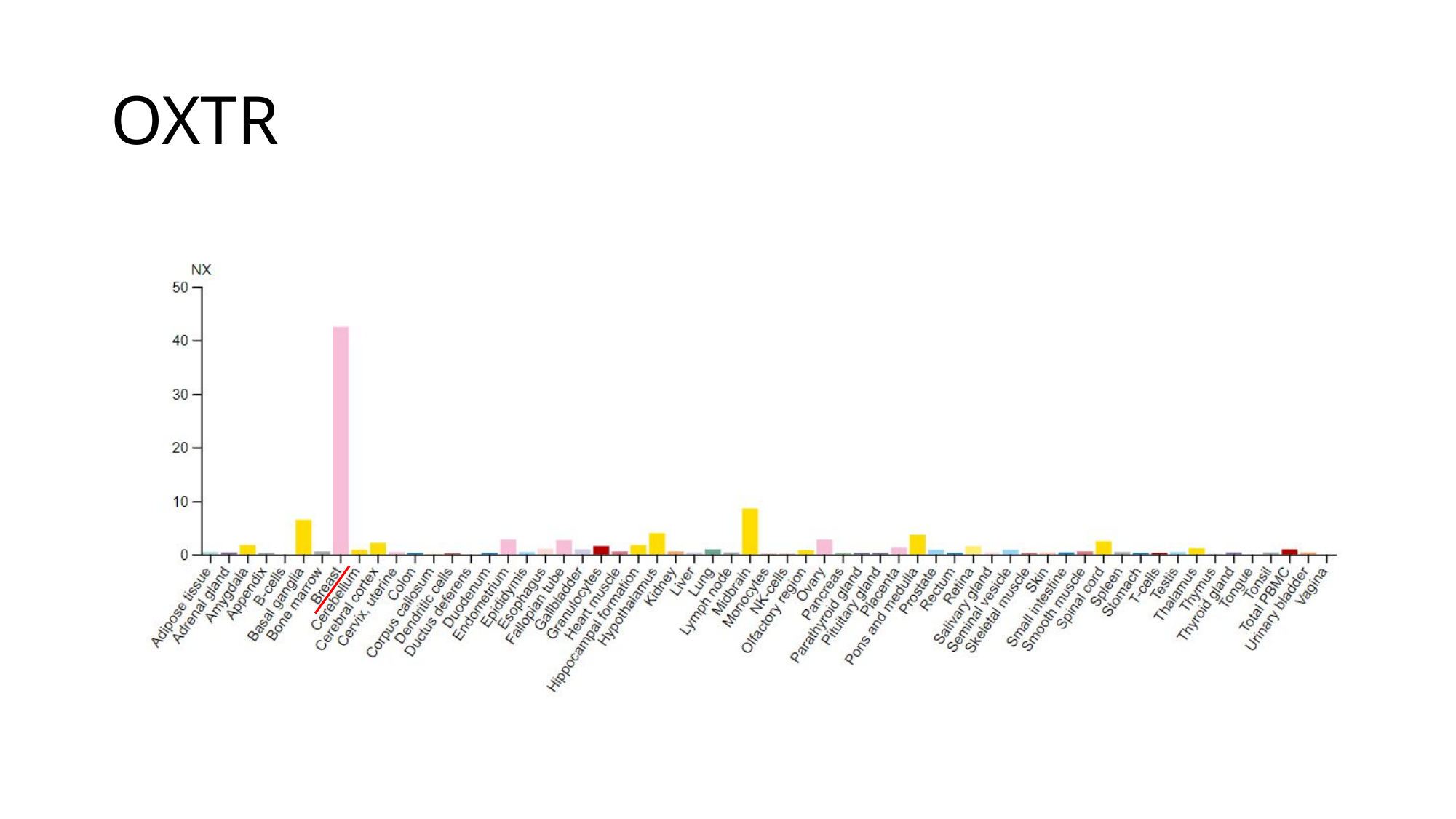

# OXTR

## Slide 9
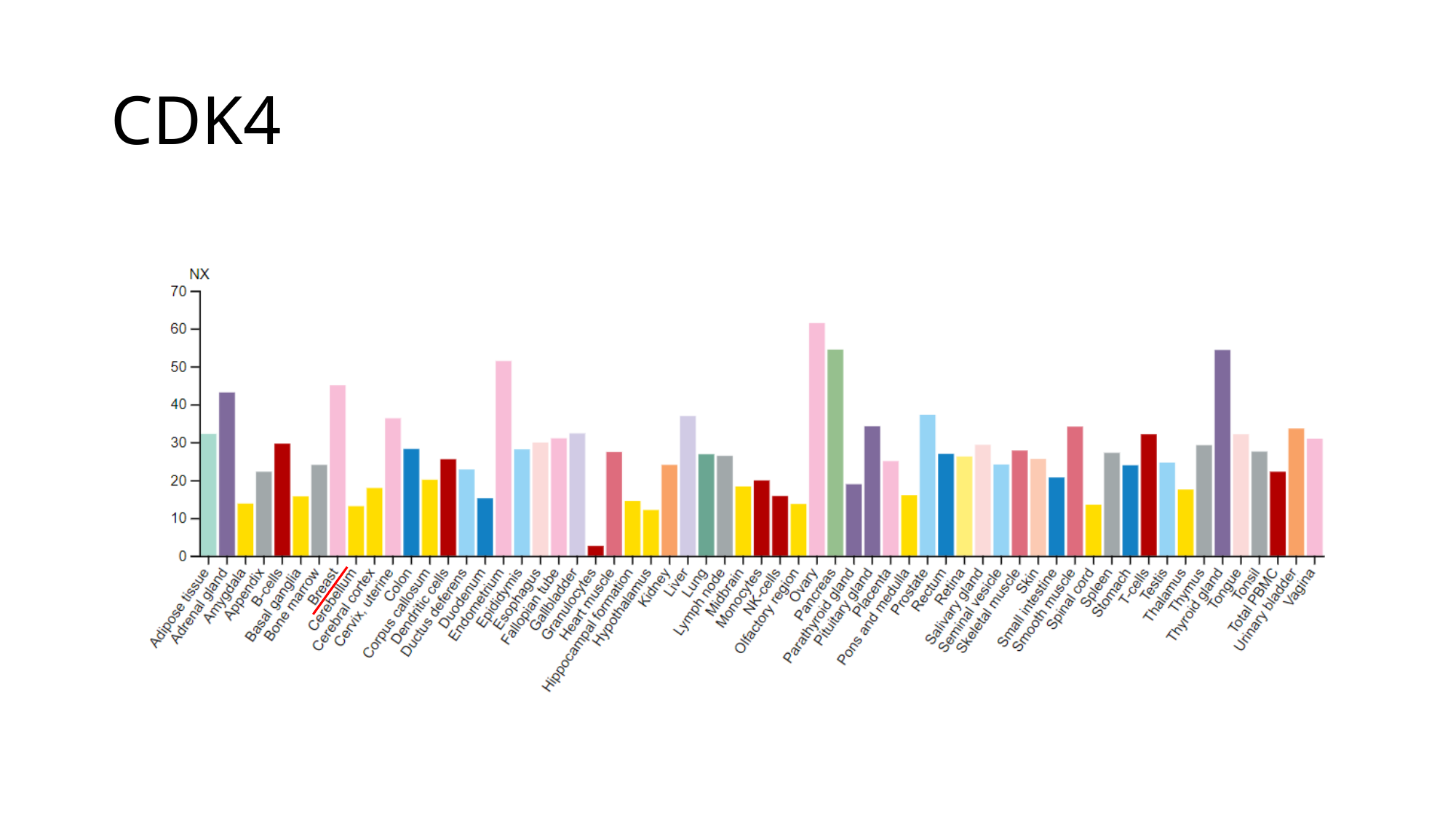

# CDK4
